# Preparation and preclinical evaluation of a ^68^Ga-labelled c(RGDfK) conjugate comprising the bifunctional chelator NODIA-Me

**DOI:** 10.1186/s41181-018-0043-2

**Published:** 2018-05-02

**Authors:** Tilman Läppchen, Jason P. Holland, Yvonne Kiefer, Mark D. Bartholomä

**Affiliations:** 1 Department of Nuclear Medicine, Medical Center – University of Freiburg, Faculty of Medicine, University of Freiburg, Hugstetterstrasse 55, D-79106 Freiburg, Germany; 2 Department of Nuclear Medicine, Inselspital, Bern University Hospital and University of Bern, Freiburgstrasse, CH-3010 Bern, Switzerland; 3 Department of Chemistry, University of Zurich, Winterthurerstrasse 190, CH-8057 Zurich, Switzerland

**Keywords:** Gallium-68, Integrins, α_v_ß_3_, PET imaging, Bifunctional chelator, NODIA-Me

## Abstract

**Background:**

We recently developed a chelating platform based on the macrocycle 1,4,7-triazacyclononane with up to three, five-membered azaheterocyclic arms for the development of ^68^Ga- and ^64^Cu-based radiopharmaceuticals. Here, a ^68^Ga-labelled conjugate comprising the bifunctional chelator NODIA-Me in combination with the α_v_ß_3_-targeting peptide c(RGDfK) has been synthesized and characterized. The primary aim was to evaluate further the potential of our NODIA-Me chelating system for the development of ^68^Ga-labelled radiotracers.

**Results:**

The BFC NODIA-Me was conjugated to c(RGDfK) by standard peptide chemistry to obtain the final bioconjugate NODIA-Me-c(RGDfK) 3 in 72% yield. Labelling with [^68^Ga]GaCl_3_ was accomplished in a fully automated, cGMP compliant process to give [^68^Ga]3 in high radiochemical yield (98%) and moderate specific activity (~ 8 MBq nmol− ^1^). Incorporation of the Ga-NODIA-Me chelate to c(RGDfK) 2 had only minimal influence on the affinity to integrin α_v_ß_3_ (IC_50_ values [^nat^Ga]3 = 205.1 ± 1.4 nM, c(RGDfK) 2 = 159.5 ± 1.3 nM) as determined in competitive cell binding experiments in U-87 MG cell line. In small-animal PET imaging and ex vivo biodistribution studies, the radiotracer [^68^Ga]3 showed low uptake in non-target organs and specific tumor uptake in U-87 MG tumors.

**Conclusion:**

The results suggest that the bifunctional chelator NODIA-Me is an interesting alternative to existing ligands for the development of ^68^Ga-labelled radiopharmaceuticals.

## Background

The transmembrane integrin α_v_ß_3_ receptor is a well-established target for imaging tumor angiogenesis. The integrin α_v_ß_3_ receptor is upregulated in activated endothelial cells of tumors undergoing angiogenesis but is not expressed in normal cells and quiescent vessel cells making it a key target for the diagnosis of malignant tumors and metastases (Hood and Cheresh 2002; Sheldrake and Patterson 2009). Besides applications in oncology, imaging of α_v_ß_3_ expression is also applied in cardiology and inflammatory diseases (Kourtzelis et al. 2017; Mandic et al. 2016).

In recent years, many peptide-based probes comprising of either one or multiple α_v_ß_3_-targeting vectors were developed for noninvasive imaging of α_v_ß_3_ expression by PET (positron emission tomography) (Gurrath et al. 1992). Reported compounds have been labelled with a range of radionuclides including fluorine-18, gallium-68 and copper-64 (Cai and Conti 2013; Chen et al. 2016; Haubner et al. 2014). For example, the radiotracers [^18^F]Fluoro-Galacto-RGD (Haubner et al. 2004), [^68^Ga/^64^Cu]Ga/Cu-NODAGA-RGD (Dumont et al. 2011), [^68^Ga/^64^Cu]Ga/Cu-DOTA-RGD (Dumont et al. 2011), [^64^Cu]Cu-AmBaSar-RGD (Cai et al. 2010) and [^68^Ga]Ga-TRAP-(RGD)_3_ (Notni et al. 2013) have demonstrated promise for imaging α_v_ß_3_. Several α_v_ß_3_-targeting radiotracers have been evaluated in clinical studies including [^18^F]Fluoro-Galacto-RGD, [^18^F]Fluoro-RGD-K5, [^18^F]FPPRGD2, [^18^F]Fluoro-fluciclatide, [^18^F]Fluoro-alfatide, [^18^F]Fluoro-alfatide II, [^68^Ga]Ga-NOTA-RGD and [^68^Ga]Ga-NOTA-PRGD2 (Cai and Conti 2013; Chen et al. 2016; Haubner et al. 2014).

We recently developed a chelating platform based on the macrocycle 1,4,7-triazacyclononane (TACN) with additional five-membered azaheterocyclic arms for the coordination of the PET radionuclides gallium-68 and copper-64 (Gotzmann et al. 2016; Schmidtke et al. 2017). Initial work revealed that these chelators are characterized by their excellent complexation properties for both radiometals. Labelling with copper-64 was achieved rapidly under very mild conditions (< 1 min incubation time, room temperature) over a pH range of 4.0 to 8.0 to give products with high specific activities (120–180 MBq nmol^− 1^). Stability studies also demonstrated that ^64^Cu-labelled complexes have high kinetic stability in vitro (Gotzmann et al. 2016). In subsequent work, we discovered that the imidazole-type ligands can also be labelled with gallium-68 (Schmidtke et al. 2017). Complexation properties were comparable to the ligand NOTA (1,4,7-triazacyclononane-1,4,7-triacetic acid) (Schmidtke et al. 2017). More recently, we described the bifunctional chelator (BFC) NODIA-Me (2-(4,7-bis((1-methyl-1H-imidazol-2-yl)methyl)-1,4,7-triazonan-1-yl)acetic acid), in which one of the methylimidazole arms was replaced with an acetic acid group. This acetic acid group served as site for the attachment of a prostate-specific membrane antigen targeting vector via peptide bond formation (Schmidtke et al. 2017). In more recent small-animal imaging and ex vivo biodistribution studies, ^64^Cu- and ^68^Ga-labelled PSMA-targeting conjugates comprising the BFC NODIA-Me specifically delineated PSMA-positive LNCaP tumors (Läppchen et al. 2018). Moreover, no significant decomplexation/transchelation of the radiometal chelate was noted in vivo, underscoring the potential use of our chelating platform for radiopharmaceutical applications.

In the present study, we sought to expand the scope of the BFC NODIA-Me for the development of ^68^Ga-based radiopharmaceuticals. Here, we report studies on a ^68^Ga-labelled α_v_ß_3_-targeting probe conjugated to the BFC NODIA-Me. The α_v_ß_3_-targeting bioconjugate NODIA-Me-c(RGDfK) was evaluated in vitro by a competitive cell binding assay, followed by small-animal PET imaging and ex vivo biodistribution studies.

## Methods

### General

Chemicals and solvents were purchased from Sigma-Aldrich and TCI Europe, and used as received. The bifunctional chelator NODIA-Me **1** was prepared as previously described (Schmidtke et al. 2017). The peptide c(RGDfK) **2** was purchased from ABX (Radeberg, Germany). The radioligand [^125^I]I-echistatin was obtained from Perkin Elmer (Boston, USA). Low resolution electrospray ionisation mass spectrometry (LR-ESI(+)-MS) was performed on a PerkinElmer Flexar SQ 300 MS Detector. Radiolabelling with [^68^Ga]GaCl_3_ was accomplished using a fully automated synthesis module (Pharmtracer, Eckert & Ziegler, Berlin, Germany) with an IGG100 generator (Eckert & Ziegler, Berlin, Germany). High performance liquid chromatography (HPLC) was performed on an Agilent 1260 Infinity System equipped with an Agilent 1200 DAD UV detector (UV detection at 220 nm) and a Raytest Ramona radiation detector (Raytest GmbH, Straubenhardt, Germany) in series. A Phenomenex Jupiter Proteo (250 × 4.60 mm) column was used for analytical HPLC. The solvent system was A = H_2_O (0.1% TFA) and B = acetonitrile (0.1% TFA). The gradient was 0–1 min 5% B, 1–20 min 40% B at a flow rate of 1 mL min^− 1^. Semi-preparative HPLC was performed on a Knauer Smartline 1000 HPLC system in combination with a Macherey Nagel VP 250/21 Nucleosil 120–5 C18 column. Semi-preparative HPLC gradient was 0–40 min 5–60% B at a flow rate of 12 mL min^− 1^. Samples were lyophilized using a Christ Alpha 1–2 LD plus lyophilizer. All instruments measuring radioactivity were calibrated and maintained in accordance with previously reported routine quality-control procedures (Zanzonico 2009). Radioactivity was measured using an Activimeter ISOMED 2010 (Nuklear-Medizintechnik, Dresden, Germany). For accurate quantification of radioactivity, experimental samples were counted for 1 min on a calibrated Perkin Elmer (Waltham, MA, USA) 2480 Automatic Wizard Gamma Counter by using a dynamic energy window of 400–600 keV for gallium-68 (511 keV emission).

### Synthesis of NODIA-Me-c(RGDfK) (3)

NODIA-Me **1** (1.25 mg, 0.003 mmol), HATU (1-[bis(dimethylamino)methylene]-1*H*-1,2,3-triazolo[4,5-b]pyridinium-3-oxide hexafluorophosphate) (2 mg, 0.005 mmol) and DIPEA (*N*,*N*-diisopropylethylamine) (2.6 mg, 4 μL, 0.02 mmol) were mixed in 500 μL anhydrous *N*,*N*-dimethylformamide (DMF) and allowed to stir for 30 min at room temperature (r.t.). Next, the peptide c(RGDfK) **2** (2 mg, 0.003 mmol) in 100 μL DMF was added and stirring was continued for additional 2 h. The solvent was removed by rotary evaporation and the residue was taken up in water/acetonitrile (50:50 *v*/v) with 0.1% trifluoroacetic acid (TFA) and purified by semi-preparative HPLC. Fractions containing the product were combined and lyophilized to give compound **3** as white powder (2.3 mg, 0.0024 mmol, 72%). LR-ESI-MS calcd m/z for C_45_H_69_N_16_O_8_ ([M + H]^+^): 961.5, found: 961.8. RP-HPLC (analytical): *t*_R_ = 11:10 min. RP-HPLC (semi-preparative): *t*_R_ = 6:10 min.

### Preparation of ^nat^Ga-NODIA-Me-c(RGDfK) ([^nat^Ga]3)

The bioconjugate NODIA-Me-c(RGDfK) **3** (500 μg, 0.52 μmol) in 250 μL H_2_O was mixed with 250 μL of metal stock solution containing 1.5 equivalents of Ga(NO_3_)_3_ and heated at 95 °C for 15 min. After cooling to r.t., the complex was purified using C_18_ Sep-Pak cartridge, which was preconditioned with 5 mL each of EtOH and H_2_O, respectively. After loading, the cartridge was washed with 2 mL H_2_O and the product was eluted using 2 mL EtOH:H_2_O (50:50 v/v). After evaporation of EtOH at ambient temperature, the remaining solution was lyophilized to give [^nat^Ga]3 (470 μg, 0.45 μmol, 88%). RP-HPLC (analytical): *t*_R_ = 14:20 min. LR-ESI-MS calcd m/z for C_45_H_68_GaN_16_O_8_ ([M]^2+^): 514.5, found: 514.9.

### Radiosynthesis of [^68^Ga]Ga-NODIA-Me-c(RGDfK) ([^68^Ga]3)

Radiolabelling of **3** with [^68^Ga]GaCl_3_ was accomplished by using the Modular-Lab PharmTracer automated synthesis module (Eckert&Ziegler, Berlin, Germany) as previously described (Schmidtke et al. 2017). An amount of 20 μg of **3** was used per labelling. The generator eluate provided ~ 350–450 MBq [^68^Ga]GaCl_3_. The radiochemical purity (RCP) was > 98% and the decay corrected radiochemical yield (RCY) was > 98%. The mean molar activity was A_m_ = 8.4 ± 2.1 MBq nmol^− 1^ (*n* = 15). RP-HPLC (analytical, radioactivity detector): *t*_R_ ([^68^Ga]**3**) = 14:30 min.

### Lipophilicity (log D_oct/PBS_) measurements

For log *D*_oct/PBS_ measurements, 1–2 MBq of [^68^Ga]**3** in 20 μL labelling buffer were added to a mixture of phosphate buffered saline (PBS) pH 7.4 (Gibco Life Science Technologies, 4 mM phosphate buffer, 0.15 M NaCl) (480 μL) and octanol (500 μL). Samples were shaken for 30 min at room temperature, centrifuged at 13,200 rpm for 5 min and 100 μL of each phase were counted using a Packard Cobra gamma counter. Experiments were performed in triplicate.

### Cell culture

U-87 MG cells (ATCC, Manassas, VA, USA) were cultured at 37 °C in a 5% CO_2_ atmosphere (Dulbecco modified Eagle medium with GlutaMAX containing 10% fetal bovine serum, 1% 10,000 U mL^− 1^ penicillin and 10,000 U mL^− 1^ streptomycin, 1% sodium-pyruvate 100 mM).

### Competitive binding assay

The binding affinity of [^nat^Ga]**3** was determined by a cell-based competitive binding assay in the human glioma cell line U-87 MG with [^125^I]I-echistatin as the radioligand as previously described (Dumont et al. 2011). Binding assays were performed in 24-well plates precoated with poly-L-lysine. Briefly, each compound at different concentrations (0–10,000 nM) was incubated for 2 h at r.t. with [^125^I]I-echistatin (30.000 cpm well^− 1^) and 2 × 10^5^ U-87 MG cells well^− 1^. After incubation, cells were washed three times with ice cold binding buffer and cell-associated activity recovered by addition of 1 M NaOH. Radioactivity was measured by a gamma counter and data fitted using non-linear regression (GraphPad Prism). Experiments were performed two times in triplicate.

### Small-animal PET imaging

All animal experiments complied with the current laws of the Federal Republic of Germany and were conducted according to German Animal welfare guidelines. Normal female athymic Balb/c nude mice (17–20 g, 4–6 weeks old) were obtained from Janvier SAS (St. Berthevin Cedex, France). Mice were provided with food and water ad libitum. U-87 MG tumors were inoculated on the right shoulder by sub-cutaneous injection of 5×10^6^ cells in a 100 μL cell suspension of a 1:1 *v*/v mixture of media with reconstituted basement membrane (GFR BD Matrigel™, Corning BV, Amsterdam, Holland).

For PET imaging studies, mice (*n* = 3) were injected with 100 μL sterile filtered phosphate buffered saline formulations pH 7.4 of [^68^Ga]**3** (7–11 MBq) by intravenous tail-vein injection and anesthetized with isoflurane (2–4% in air) 5–10 min prior image acquisition. PET imaging was performed on a Focus 120 microPET scanner at 1 h after administration. Data were acquired 1 h post administration in list mode. Reconstruction was performed using unweighted OSEM2D. Image analysis was performed using AMIDE. Image counts per second per voxel (cps/voxel) were calibrated to activity concentrations (Bq mL^− 1^) by measuring a 3.5 cm cylinder phantom filled with a known concentration of radioactivity. For data analysis, it was explicitly assumed that the density of tissue equals 1.0 g cm^− 3^, hence the reported units of %IA g^− 1^ are identical to %IA cm^− 3^. Specificity of [^68^Ga]**3** was confirmed by competitive inhibition (blocking) co-injecting the peptide c(RGDfK) **2** (5 mg kg^− 1^ = ~ 100 nmol mouse^− 1^; *n* = 3) in approximately 100fold excess compared to the radiotracer.

### Ex vivo biodistribution

For each compound, a total of five animals were injected with [^68^Ga]**3** (7–11 MBq) in 100 μL sterile filtered phosphate buffered saline via a tail vein. At 1 h p.i., animals were sacrificed by isoflurane anesthesia. Organs of interest were dissected, weighed and assayed for radioactivity in a gamma counter. The percent injected activity per gram (%IA g^− 1^) for each tissue was calculated by comparison of the tissue counts to a standard sample prepared from the injectate. Specificity of [^68^Ga]**3** was determined by co-injection of the peptide c(RGDfK) **2** (5 mg kg^− 1^ = ~ 100 nmol mouse^− 1^).

## Results and discussion

### Bioconjugate synthesis

**Scheme 1:**
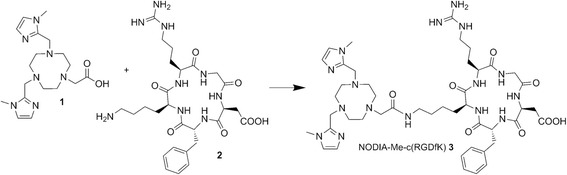
Synthesis of NODIA-Me-c(RGDfK) **3**. HATU, DIPEA, DMF, r.t., 72%

The bifunctional chelator NODIA-Me **1** bearing an acetic acid residue for the covalent attachment of appropriate targeting vectors was successfully conjugated to the peptide c(RGDfK) **2** in DMF using HATU as coupling reagent (Scheme 1). The final bioconjugate **3** was obtained in 72% yield after purification by semi-preparative RP-HPLC. The identity and purity of compound **3** (> 98%) was determined by mass spectrometry and analytical RP-HPLC.

### Radiochemistry

Radiosynthesis of [^68^Ga]**3** was performed using an automated synthesis module by heating 20 μg of compound **3** with [^68^Ga]GaCl_3_(aq.) at 95 °C for 10 min as previously described (Schmidtke et al. 2017). The RCP and the decay corrected RCY for [^68^Ga]**3** were measured to > 98% with mean specific activities of ~ 8 MBq nmol^− 1^. The identity of [^68^Ga]**3** was confirmed by analytical HPLC using the non-radioactive reference compound.

### Lipophilicity

The radiolabelled conjugate [^68^Ga]**3** is highly hydrophilic with a log *D*_oct/PBS_ value of − 3.89 ± 0.02. Interestingly, in a series of c(RGDfK) based radiopharmaceuticals with different bifunctional chelators that give gallium complexes of different overall charge, [^68^Ga]**3** with a positively charged metal chelate was more hydrophilic than [^68^Ga]Ga-NODAGA-c(RGDfK) or [^68^Ga]Ga-DOTA-c(RGDfK), which have a neutral and negative overall charge on the metal chelate (log *D* values: − 3.27 ± 0.01 and − 2.86 ± 0.01), respectively (Dumont et al. 2011).

### Competitive binding assay

**Fig. 1:**
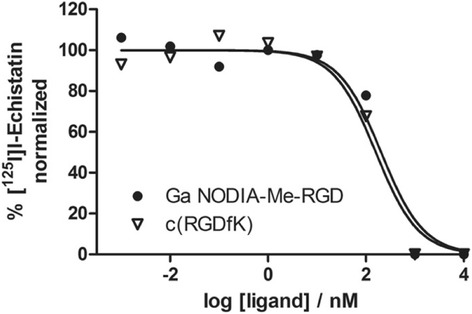
Inhibition of [^125^I]I-echistatin binding to integrin α_v_ß_3_ on U-87 MG cells by c(RGDfK) **2** (IC_50_ = 159.5 ± 1.3 nM) and ^nat^Ga-NODIA-Me-c(RGDfK) [^nat^Ga]**3** (IC_50_ = 205.1 ± 1.4 nM) (*n* = 2 in triplicate, mean ± SD)

The binding affinity of [^nat^Ga]**3** was compared to c(RGDfK) **2** in a competitive binding assay on α_v_ß_3_-positive U-87 MG cells using [^125^I]I-echistatin as radioligand. Both compounds inhibited the binding of [^125^I]I-echistatin in a dose dependent manner. The IC_50_ values for **2** and [^nat^Ga]**3** were determined to IC_50_ = 159.5 ± 1.3 nM and IC_50_ = 205.1 ± 1.4 nM, which are in accordance to previously reported IC_50_ values (Dumont et al. 2011). Introduction of the metal chelate had only a minimal effect on receptor binding resulting in a slightly lower affinity of [^nat^Ga]**3** compared to peptide **2**. Corresponding inhibition curves are given in Fig. 1.

### In vivo studies

In our efforts to demonstrate the applicability of our novel chelating system for radiopharmaceutical applications, we assessed the stability and pharmacokinetic profile of [^68^Ga]**3** by small-animal experiments.

### Ex vivo biodistribution

Biodistribution data for [^68^Ga]**3** are presented in Table 1. For comparison, equivalent data taken from the literature are also given for compounds [^68^Ga]Ga-NODAGA-c(RGDfK) and [^68^Ga]Ga-DOTA-c(RGDfK) (Dumont et al. 2011). The primary difference between these three compounds is the change in chelator. The highest activity accumulation of [^68^Ga]**3** among all tissues at 1 h p.i. was seen for the α_v_ß_3_-positive U-87 MG tumors with 2.10 ± 0.09 %IA g^− 1^. Despite differences in the tumor models used, accumulation of [^68^Ga]**3** in U-87 MG xenografts is comparable to that of previously reported ^68^Ga-labelled RGD peptides in α_v_ß_3_-expressing tumors (Boros et al. 2012; Ferreira et al. 2012; Pohle et al. 2012). In comparison to [^68^Ga]Ga-NODAGA-c(RGDfK) and [^68^Ga]Ga-DOTA-c(RGDfK), which have previously been evaluated under similar conditions, tumor uptake of [^68^Ga]**3** was somewhat lower than that of [^68^Ga]Ga-NODAGA-c(RGDfK) and [^68^Ga]Ga-DOTA-c(RGDfK) (Table 1) (Dumont et al. 2011). Tumor uptake was significantly reduced by 86% (*P* value < 0.0001) by co-injection of c(RGDfK), indicating receptor specific binding in tumor tissue. Accumulation in the kidneys and the liver was low considering the positively charged NODIA-Me metal chelate because positively charged compounds might be retained in both organs (Dearling et al. 2013; Dearling et al. 2015; Sprague et al. 2007). In previous animal studies of our ^64^Cu-labelled chelators (without a targeting vector), substantial uptake and retention of activity was observed in the kidneys by small-animal PET imaging (Gotzmann et al. 2016). This kidney accumulation of radioactivity was attributed to the positive overall charge of the metal complexes. In the present study, [^68^Ga]**3** exhibited the lowest kidney uptake compared to the previously described compounds [^68^Ga]Ga-NODAGA-c(RGDfK) and [^68^Ga]Ga-DOTA-c(RGDfK). Obviously, the pharmacokinetics of [^68^Ga]**3** are mainly determined by the peptidic targeting vector. However, all three compounds make use of different bifunctional chelators that produce ^68^Ga complexes of different shape, overall charge and lipophilicity. In contrast to DOTA and NODAGA, the chelator NODIA-Me **1** does not possess any charge compensating donor atoms and forms complexes with [^68^Ga]Ga^3+^ of an overall charge of either 2+ or 3+, whether the remaining coordination site of the gallium octahedron is occupied by a monodentate ligand such as hydroxide or chloride or not. While we have been able to characterize the exchange of chloride vs. hydroxide for the bioconjugate NODIA-Me-PSMA (Schmidtke et al. 2017), we neither observed such an exchange in a more recent study with another PSMA-targeting conjugate (Läppchen et al. 2018) nor in the present study. On the other hand, [^68^Ga]Ga-NODAGA-c(RGDfK) and [^68^Ga]Ga-DOTA-c(RGDfK) give complexes that have an overall charge of 0 and − 1, respectively (Dumont et al. 2011). Obviously, the differences of the metal chelates resulted in different pharmacokinetic profiles of the tracers underlining the influence of the metal binding moiety on the biological properties of the final radiopharmaceutical. A similar trend as found for the kidneys was observed for the liver with [^68^Ga]**3** exhibiting the lowest liver uptake in this series. On the other hand, the blood activity of [^68^Ga]**3** with 0.54 ± 0.08 %IA g^− 1^ was comparable to that of [^68^Ga]Ga-DOTA-c(RGDfK) with 0.38 ± 0.07 %IA g^− 1^ and slightly higher than that of [^68^Ga]Ga-NODAGA-c(RGDfK) with 0.16 ± 0.03 %IA g^− 1^. Accumulation of [^68^Ga]**3** at 1 h p.i. in all other organs was low (< 1 %IA g^− 1^). Interestingly, [^68^Ga]**3** exhibited lower uptake in the spleen, intestine, stomach and lung compared to [^68^Ga]Ga-NODAGA-c(RGDfK) and [^68^Ga]Ga-DOTA-c(RGDfK). Despite the lower tumor uptake, the tumor-to-tissue ratios of [^68^Ga]**3** for the liver, kidneys and muscle are comparable to those of [^68^Ga]Ga-NODAGA-c(RGDfK) and [^68^Ga]Ga-DOTA-c(RGDfK) (Table 1).

**Table 1:** Ex vivo biodistribution of [^68^Ga]**3** in mice bearing α_v_ß_3_-positive U-87 MG tumors at 1 h p.i. along with blocking studies in comparison to [^68^Ga]Ga-NODAGA-c(RGDfK) and [^68^Ga]Ga-DOTA-c(RGDfK) (data taken from ref. Dumont et al. 2011). Data are expressed as %IA g^− 1^ and represent mean ± SD (*n* = 5)

Organ	[^68^Ga]3		[^68^Ga]Ga-NODAGA-c(RGDfK)	[^68^Ga]Ga-DOTA-c(RGDfK)
	1 h	1 h blockade	1 h	1 h
Blood	0.54 ± 0.08	0.50 ± 0.03	0.16 ± 0.03	0.38 ± 0.07
Heart	0.31 ± 0.05	0.16 ± 0.03	0.33 ± 0.07	0.35 ± 0.08
Lung	0.62 ± 0.09	0.33 ± 0.06	0.80 ± 0.07	0.87 ± 0.12
Spleen	0.73 ± 0.05	0.34 ± 0.05	1.73 ± 0.44	1.34 ± 0.21
Liver	1.02 ± 0.13	0.37 ± 0.20	1.86 ± 0.23	1.60 ± 0.27
Pancreas	0.21 ± 0.01	0.09 ± 0.07	0.23 ± 0.08	0.29 ± 0.05
Stomach	0.54 ± 0.13	0.12 ± 0.02	1.40 ± 0.34	1.50 ± 0.36
Intestine	0.31 ± 0.01	0.13 ± 0.01	1.83 ± 0.51	1.79 ± 0.30
Kidney	1.65 ± 0.12	0.87 ± 0.16	1.98 ± 0.51	2.24 ± 0.34
Muscle	0.18 ± 0.01	0.09 ± 0.01	0.49 ± 0.29	0.29 ± 0.04
Bone	0.36 ± 0.06	0.14 ± 0.04	0.45 ± 0.21	0.35 ± 0.05
Tumor	2.10 ± 0.09	0.29 ± 0.16	5.19 ± 1.45	3.47 ± 0.78
Tumor-to-blood	3.91 ± 0.43		27.67 ± 7.01	9.24 ± 1.12
Tumor-to-kidney	1.27 ± 0.04		2.64 ± 0.31	1.57 ± 0.14
Tumor-to-liver	2.08 ± 0.18		2.75 ± 0.31	2.25 ± 0.37
Tumor-to-muscle	11.69 ± 0.29		12.80 ± 5.25	12.37 ± 1.81

The specificity of [^68^Ga]**3** for integrin α_v_ß_3_ was confirmed in blocking studies by co-injection of an ~100fold excess c(RGDfK), which resulted in a significant reduction of tracer uptake in all tissues (*P* values < 0.05 for all tissues). This is in accordance with the literature where α_v_ß_3_ imaging probes demonstrate low but blockable uptake in normal tissues (Chen et al. 2004; Decristoforo et al. 2008; Dijkgraaf et al. 2011; Li et al. 2007; Wei et al. 2009).

### Small-animal PET imaging

**Fig. 2:**
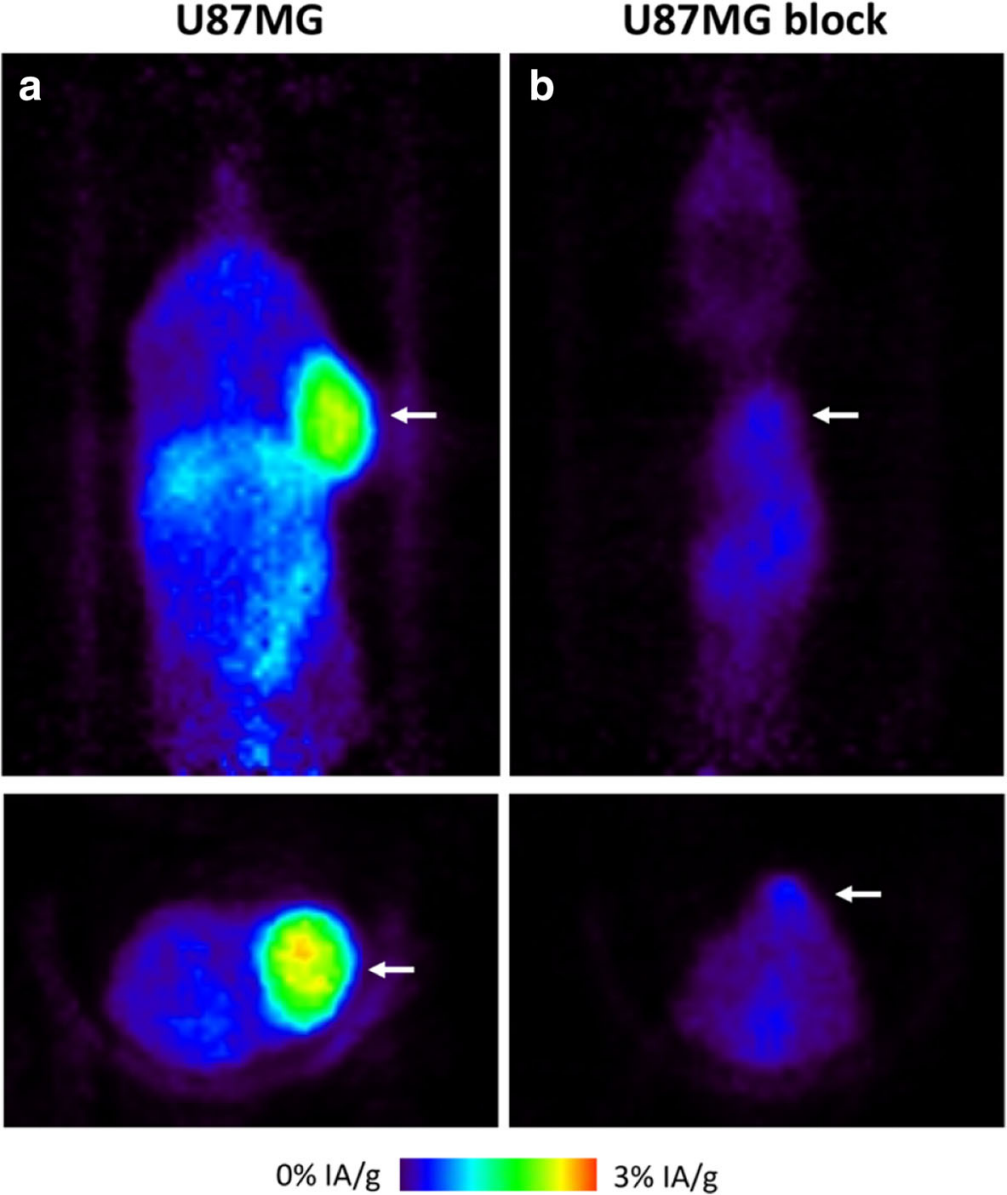
**a** Representative coronal (top) and transverse (bottom) maximum intensity projections (MIPs) of [^68^Ga]**3** at 1 h p.i. in U-87 MG tumor bearing mice. **b** Blocking studies confirmed the specificity of [^68^Ga]**3** for α_v_ß_3_ expression. White arrows indicate the tumors

In addition to the ex vivo biodistribution studies, the distribution profile of [^68^Ga]**3** was also assessed 1 h after administration by small-animal PET imaging. Corresponding transverse and coronal maximum intensity projections of [^68^Ga]**3** in α_v_ß_3_ xenograft bearing mice along with blocking studies are given Fig. 2. The results of the biodistribution study were confirmed by PET imaging. The α_v_ß_3_ positive U-87 MG tumors as well as the liver and the kidneys were clearly visible on the PET images. The tumor uptake of [^68^Ga]**3** was determined to 2.48 ± 0.14 %IA g^− 1^. The differences in blood and tissue activity versus tumor activity between [^68^Ga]**3**, [^68^Ga]Ga-NODAGA-c(RGDfK) and [^68^Ga]Ga-DOTA-c(RGDfK) gave a tumor-to-background ratio of 6.76 for [^68^Ga]**3** that was lower than that of [^68^Ga]Ga-NODAGA-c(RGDfK) (11.97) but about 2-fold higher than that of [^68^Ga]Ga-DOTA-c(RGDfK) (3.28) (Dumont et al. 2011). The specificity of [^68^Ga]**3** was confirmed in blockade studies by co-injecting c(RGDfK) shown in Fig. 2b, which resulted in a significant reduction of tumor accumulation (0.67 ± 0.10 %IA g^− 1^) (*P* value < 0.0001) and reduced uptake in all other organs.

## Conclusions

In this work, we successfully developed a ^68^Ga-labelled bioconjugate comprising our novel chelator NODIA-Me for imaging the α_v_ß_3_ integrin receptor. The final bioconjugate was readily obtained in a single reaction step by standard peptide chemistry in good yields. The resulting bioconjugate was labelled with [^68^Ga]GaCl_3_ in a cGMP compliant process in high yields and moderate specific activity. Introduction of the novel metal chelate to the peptide c(RGDfK) had only a minimal impact on receptor binding. U-87 MG tumors overexpressing the α_v_ß_3_ integrin receptor were specifically delineated in ex vivo biodistribution and small-animal PET imaging studies by the corresponding ^68^Ga-labelled bioconjugate. Uptake of the novel tracer in non-target tissues was low providing acceptable tumor-to-background ratios. Even though tumor uptake and tumor-to-background ratios were lower compared to [^68^Ga]Ga-NODAGA-c(RGDfK), the low uptake in non-target tissues indicates kinetically stable complexation of gallium-68 in the bifunctional chelator NODIA-Me. Altogether, our results demonstrate that our novel chelating system is an interesting alternative to existing bifunctional chelators such as DOTA and NODAGA for ^68^Ga-based radiopharmaceuticals.

## Abbreviations

%IA g^-1^: Percent of injected activity per gram
BFC: Bifunctional chelator
DIPEA: *N*,*N*-diisopropylethylamine
DMF: *N*,*N*-dimethylformamide
DOTA: 1,4,7,10-tetraazacyclododecane-1,4,7,10-tetraacetic acid
HATU: 1-[bis(dimethylamino)methylene]-1*H*-1,2,3-triazolo[4,5-b]pyridinium-3-oxide hexafluorophosphate
MIP: Maximum intensity projection
NODAGA: 1,4,7-triazacyclononane -1,4-diacetic acid-7-glutaric acid
NODIA-Me: 2-(4,7-bis((1-methyl-1H-imidazol-2-yl)methyl)-1,4,7-triazonan-1-yl)acetic acid
NOTA: 1,4,7-triazacyclononane-1,4,7-triacetic acid
PSMA: Prostate specific membrane antigen
RCP: Radiochemical purity
RCY: Radiochemical yield
TACN: 1,4,7-triazacyclononane
TFA: Trifluoroacetic acid (TFA)

## References

[CR1] Boros E, Ferreira CL, DTT Y, Gill RK, Price EW, Adam MJ, Orvig C (2012). RGD conjugates of the H(2)dedpa scaffold: synthesis, labeling and imaging with Ga-68. Nucl Med Biol.

[CR2] Cai HC, Conti PS (2013). RGD-based PET tracers for imaging receptor integrin alpha(v)beta(3) expression. J Label Compd Radiopharm.

[CR3] Cai HC, Li ZB, Huang CW, Shahinian AH, Wang H, Park R, Conti PS (2010). Evaluation of Copper-64 labeled AmBaSar conjugated cyclic RGD peptide for improved MicroPET imaging of integrin alpha(v)beta(3) expression. Bioconjug Chem.

[CR4] Chen HJ, Niu G, Wu H, Chen XY (2016). Clinical application of radiolabeled RGD peptides for PET imaging of integrin alpha(v)beta(3). Theranostics.

[CR5] Chen XY, Hou YP, Tohme M, Park R, Khankaldyyan V, Gonzales-Gomez I, Bading JR, Laug WE, Conti PS (2004). Pegylated Arg-Gly-Asp peptide: Cu-64 labeling and PET imaging of brain tumor alpha(v)beta(3)-integrin expression. J Nucl Med.

[CR6] Dearling JL, Paterson B, Dunning P, Snay E, Treves ST, Voss S (2013). The effect of chelator charge on the biodistribution of engineered antibodies labeled with 64Cu. J Nucl Med Meeting Abstracts.

[CR7] Dearling JL, Paterson BM, Akurathi V, Betanzos-Lara S, Treves ST, Voss SD, White JM, Huston JS, Smith SV, Donnelly PS, Packard AB (2015). The ionic charge of copper-64 complexes conjugated to an engineered antibody affects biodistribution. Bioconjug Chem.

[CR8] Decristoforo C, Gonzalez IH, Carlsen J, Rupprich M, Huisman M, Virgolini I, Wester HJ, Haubner R (2008). (68)Ga- and (111)In-labelled DOTA-RGD peptides for imaging of alpha v beta 3 integrin expression. Eur J Nucl Med Mol Imaging.

[CR9] Dijkgraaf I, Yim CB, Franssen GM, Schuit RC, Luurtsema G, Liu SA, Oyen WJG, Boerman OC (2011). PET imaging of alpha(v)beta(3) integrin expression in tumours with Ga-68-labelled mono-, di- and tetrameric RGD peptides. Eur J Nucl Med Mol Imaging.

[CR10] Dumont RA, Deininger F, Haubner R, Maecke HR, Weber WA, Fani M (2011). Novel Cu-64- and Ga-68-labeled RGD conjugates show improved PET imaging of alpha(v)beta(3) integrin expression and facile radiosynthesis. J Nucl Med.

[CR11] Ferreira CL, Yapp DTT, Mandel D, Gill RK, Boros E, Wong MQ, Jurek P, Kiefer GE (2012). Ga-68 small peptide imaging: comparison of NOTA and PCTA. Bioconjug Chem.

[CR12] Gotzmann C, Braun F, Bartholomä MD (2016). Synthesis, 64Cu-labeling and PET imaging of 1,4,7-triazacyclononane derived chelators with pendant azaheterocyclic arms. RSC Adv.

[CR13] Gurrath M, Muller G, Kessler H, Aumailley M, Timpl R (1992). Conformation activity studies of rationally designed potent antiadhesive Rgd peptides. Eur J Biochem.

[CR14] Haubner R, Kuhnast B, Mang C, Weber WA, Kessler H, Wester HJ, Schwaiger M (2004). [F-18]Galacto-RGD: synthesis, radiolabeling, metabolic stability, and radiation dose estimates. Bioconjug Chem.

[CR15] Haubner R, Maschauer S, Prante O. PET radiopharmaceuticals for imaging integrin expression: tracers in clinical studies and recent developments. Biomed Res Int. 2014; 10.1155/2014/871609. 10.1155/2014/871609 PMC4072020 25013808

[CR16] Hood JD, Cheresh DA (2002). Role of integrins in cell invasion and migration. Nat Rev Cancer.

[CR17] Kourtzelis I, Mitroulis I, von Renesse J, Hajishengallis G, Chavakis T. From leukocyte recruitment to resolution of inflammation: the cardinal role of integrins. J Leukoc Biol. 2017; 10.1189/jlb.3MR0117-024R. 10.1189/jlb.3MR0117-024R PMC5557641 28292945

[CR18] Läppchen T, Kiefer Y, Holland JP, Bartholomä MD. In vitro and in vivo evaluation of the bifunctional chelator NODIA-Me in combination with a prostate-specific membrane antigen targeting vector. Nucl Med Biol. 2018; 10.1016/j.nucmedbio.2018.03.002. 10.1016/j.nucmedbio.2018.03.002 29571066

[CR19] Li ZB, Cai WB, Cao QZ, Chen K, Wu ZH, He LN, Chen XY (2007). 64Cu-labeled tetrameric and octameric RGD peptides for small-animal PET of tumor alpha(v)beta(3) integrin expression. J Nucl Med.

[CR20] Mandic L, Traxler D, Gugerell A, Zlabinger K, Lukovic D, Pavo N, Goliasch G, Spannbauer A, Winkler J, Gyongyosi M (2016). Molecular imaging of angiogenesis in cardiac regeneration. Curr Cardiovasc Imaging Rep.

[CR21] Notni J, Pohle K, Wester HJ (2013). Be spoilt for choice with radiolabelled RGD peptides: preclinical evaluation of Ga-68-TRAP(RGD)(3). Nucl Med Biol.

[CR22] Pohle K, Notni J, Bussemer J, Kessler H, Schwaiger M, Beer AJ (2012). Ga-68-NODAGA-RGD is a suitable substitute for F-18-Galacto-RGD and can be produced with high specific activity in a cGMP/GRP compliant automated process. Nucl Med Biol.

[CR23] Schmidtke A, Läppchen T, Weinmann C, Bier-Schorr L, Keller M, Kiefer Y, Holland JP, Bartholomä MD. Gallium complexation, stability, and bioconjugation of 1,4,7-triazacyclononane derived chelators with azaheterocyclic arms. Inorg Chem. 2017; 10.1021/acs.inorgchem.7b01129. 10.1021/acs.inorgchem.7b01129 28742337

[CR24] Sheldrake HM, Patterson LH (2009). Function and antagonism of beta3 integrins in the development of cancer therapy. Curr Cancer Drug Targets.

[CR25] Sprague JE, Peng Y, Fiamengo AL, Woodin KS, Southwick EA, Weisman GR, Wong EH, Golen JA, Rheingold AL, Anderson CJ (2007). Synthesis, characterization and in vivo studies of Cu(II)-64-labeled cross-bridged tetraazamacrocycle-amide complexes as models of peptide conjugate imaging agents. J Med Chem.

[CR26] Wei L, Ye Y, Wadas TJ, Lewis JS, Welch MJ, Achilefu S, Anderson CJ (2009). Cu-64-labeled CB-TE2A and diamsar-conjugated RGD peptide analogs for targeting angiogenesis: comparison of their biological activity. Nucl Med Biol.

[CR27] Zanzonico P (2009). Routine quality controlof clinical nucliear medicine instrumentation: a brief review. J Nucl Med.

